# miR-466a Targeting of TGF-β2 Contributes to FoxP3^+^ Regulatory T Cell Differentiation in a Murine Model of Allogeneic Transplantation

**DOI:** 10.3389/fimmu.2018.00688

**Published:** 2018-04-09

**Authors:** William Becker, Mitzi Nagarkatti, Prakash S. Nagarkatti

**Affiliations:** Department of Pathology, Microbiology and Immunology, University of South Carolina School of Medicine, Columbia, SC, United States

**Keywords:** allotransplantation, epigenetics, microRNAs, transforming growth factor beta 2, T regulatory cells

## Abstract

The promise of inducing immunological tolerance through regulatory T cell (Treg) control of effector T cell function is crucial for developing future therapeutic strategies to treat allograft rejection as well as inflammatory autoimmune diseases. In the current study, we used murine allograft rejection as a model to identify microRNA (miRNA) regulation of Treg differentiation from naïve CD4 cells. We performed miRNA expression array in CD4^+^ T cells in the draining lymph node (dLN) of mice which received syngeneic or allogeneic grafts to determine the molecular mechanisms that hinder the expansion of Tregs. We identified an increase in miRNA cluster 297-669 (C2MC) after allogeneic transplantation, in CD4^+^ T cells, such that 10 of the 27 upregulated miRNAs were all from this cluster, with one of its members, mmu-miR-466a-3p (miR-466a-3p), targeting transforming growth factor beta 2 (TGF-β2), as identified through reporter luciferase assay. Transfection of miR-466a-3p in CD4^+^ T cells led to a decreased inducible FoxP3^+^ Treg generation while inhibiting miR-466a-3p expression through locked nucleic acid resulting in increased Tregs and a reduction in effector T cells. Furthermore, *in vivo* inhibition of miR-466a-3p in an allogeneic skin-graft model attenuated T cell response against the graft through an increase in TGF-β2. TGF-β2 was as effective as TGF-β1 at both inducing Tregs and through adoptive transfer, mitigating host effector T cell response against the allograft. Together, the current study demonstrates for the first time a new role for miRNA-466a-3p and TGF-β2 in the regulation of Treg differentiation and thus offers novel avenues to control inflammatory disorders.

## Introduction

Organ transplantation is a life-saving, ultimate resort for people undergoing end-stage organ failure. Thanks to an armamentarium of immunosuppressive drugs, graft loss due to acute rejection is rare; however, chronic allograft failure persists, and immunosuppression leaves patients vulnerable to infection, heart disease, nephrotoxicity, and malignancy, among others (1, 2). Exciting developments in the generation of *ex vivo* expanded regulatory T cells (Tregs) are promising candidates for suppressing graft rejection *sans* global immunosuppression (3–6). However, increased attention is needed into the mechanisms that dictate and control the generation of antigen-specific Tregs, to prevent them from reverting to a pro-inflammatory phenotype once they are introduced into the diverse cytokine milieu found *in vivo*. As shown by others, there exist opportunities for Treg differentiation into T helper (Th)1 and Th17 lymphocytes during inflammation (7, 8). In an inflammatory environment, Tregs may undergo reprogramming, wherein the Treg-specific demethylation region, which is constitutively demethylated in Tregs, may become methylated, or partially methylated in the case of peripheral Tregs (pTregs), leading to a loss in Foxp3 expression and immunosuppressive activity, conferring an acquired proclivity for graft destruction in the reprogrammed cells (9, 10). Investigations in recent years have delved into the factors dictating the differentiation (11, 12), generation (12, 13), and functions (13–16) of Treg cells. Nevertheless, there remains a need for an increased attention into the factors that can confer stable Treg-suppressive activity.

MicroRNAs (miRNAs) are one of the critical players of T cell function and plasticity (17–20). miRNAs are a group of, short, single-stranded, ~21 nucleotide-long RNA sequences that bind to the 3′ untranslated region (UTR) of target mRNAs through a six to eight nucleotide “seed sequence,” generally leading to the degradation of target mRNA or the inhibition of translation (19). Indeed, miRNAs have been found to be heavily influential in several areas of Treg biology, such as the effect of miR-155 on Treg fitness (15), miR-146a on Treg control of Th suppression, specifically Th1 responses (16, 21) and miR-21’s role in Treg expansion (22, 23). However, there remains a paucity of information concerning what miRNAs hinder Treg generation in inflammatory models. One factor that is critical to the development of FoxP3^+^ Treg cells is transforming growth factor-beta 1 (TGF-β1). At first contentious, many studies have since highlighted an indispensable role for TGF-β1 in the differentiation and generation of Tregs. Both thymic intra-medullary naïve CD4 cells (CD4^+^CD8^−^CD25^−^), and peripheral naïve CD4 cells can be differentiated into FoxP3-expressing Tregs after TCR stimulation in the presence of TGF-β1 (24, 25).

Because allograft rejection is a robust T cell-mediated immune response involving the activation of a large proportion of T cells that are alloreactive, we used that as a model to study how Tregs are silenced. To that end, we performed the expression profiling of miRNAs in CD4^+^ T cells found in the draining lymph node (dLN) of mice that received tail skin allografts to identify miRNA that influence the generation of Tregs. Our results demonstrate that a cluster of miRNA is upregulated after alloantigen exposure, specifically in dLN CD4^+^ T cells, that act to suppress TGF-β2, resulting in a decreased Treg generation and increased inflammation. The current study suggests a unique role for TGF-β2 in the regulation of Tregs and therefore opens new avenues to treat not only allograft rejection but other inflammatory disorders.

## Materials and Methods

### Animals

The University of South Carolina Institutional Animal Care and Use Committee approved all experiments. All mice were housed at the AAALAC-accredited animal facility at the University of South Carolina, School of Medicine (Columbia, SC, USA), and given *ad libitum* access to water and normal chow diet. Female C57BL/6 (H-2^b^ wild-type, BL6) and C_3_H (H-2^k^, C_3_H) mice, aged 8–12 weeks, with an average weight of 20 g, were obtained from Jackson Laboratories (Bar Harbor, ME, USA). C57BL/6 FoxP3^GFP^ mice were bred and maintained in-house. The number of mice for each experimental cohort is described in the figure legends. Each experiment was repeated at least twice, and in many cases, three or four times.

### Skin Transplant, Locked Nucleic Acid (LNA)-Based Treatment, and Adoptive-Induced Treg (iTreg) Transfer

Transplantation of tail skin from donor (C_3_H, allograft; C57BL/6, syngeneic graft) to recipient C57BL/6 mice was carried out as described previously (26). Skin grafts were obtained by excising the tail from donor mice and splitting the tail into equivalently sized ~1 × 1 cm^2^ grafts. Recipient mice were anesthetized by an intraperitoneal (i.p.) injection of ketamine (80 mg/kg) and xylazine (12 mg/kg) (Southern Anesthesia & Surgical, Columbia, SC, USA) in molecular-grade water. Upon sufficient anesthetic depth, mice were shaved and ~1 × 1 cm^2^ graft beds were made using curved scissors on the dorsal lateral surface. Donor skin grafts were placed onto the graft beds and mice were bandaged. Mice were monitored and kept in bandages for 7–9 days following skin transplantation surgery. In studies using LNA-based miRNA inhibitor (anti-miR-466a-3p, Exiqon), the LNA (10 mg/kg) was injected i.p. to graft-recipient mice 1 day before skin transplant and then every third day after that until termination of the study. For studies involving expanded iTregs, these cells were cultured as described below, sorted for CD4^+^, FoxP3-GFP expression using BD FACSAria II, and 1 × 10^6^ iTregs were adoptively transferred 1 day before skin grafting. For graft rejection scoring, mice were scored as +/+, viable graft; +/−, partially rejected the graft (>50% scabbed over or necrotic, or >50% reduction in graft size); or −/−, fully rejected the graft (>80% necrotic). For depicting graft survival, +/+ and +/− skin grafts were considered viable, and −/− skin grafts were considered nonviable. The log-rank method was used to determine differences in graft survival.

### Target Prediction and Luciferase Reporter Assays

Relevant targets for miR-466a-3p and other miRNAs were investigated by cross-referencing predictions from TargetScan Mouse 6.2 software using a context + score threshold greater than −0.02 and microRNA.org using a mirSVR score between −1.2 and −0.2. The 3′ UTR of candidate gene targets or mutated control was purchased from Integrated DNA Technologies and cloned immediately downstream of luciferase in the pMiReport vector (Promega, Madison, WI, USA). The insertion of candidate mRNAs was verified through PCR and agarose gel electrophoresis. For luciferase assays, 2.5 × 10^5^ EL-4 cells were plated in 24-well plates for 24 h and subsequently transfected with either luciferase reporter constructs, together with miR-466a-3p mimics, or the negative scramble control (Qiagen, Valencia, CA, USA) using lipofectamine 3000 (Life Technologies). At 48 h post transfection, dual luciferase assay system (Promega, Madison, WI, USA) was used to detect luciferase activity. Normalized data were calculated as the quotient of Renilla/firefly luciferase activities. The experiments were performed in duplicate and repeated at least three times.

### Cell Culture

Cells were cultured in a sterile incubator that was maintained at 37°C and 5% CO_2_. EL-4 cells were cultured in DMEM supplemented with 10% fetal bovine serum, 100 U/mL penicillin, and 100 U/mL streptomycin. Primary cells were cultured in complete RPMI supplemented with 10% FBS, 100 U/mL penicillin, 100 U/mL streptomycin (both Gibco), 10 mM HEPES (Gibco, Paisley, UK), and 50 µM β-mercaptoethanol (Sigma-Aldrich, Gillingham, UK) (complete medium).

### Treg Polarization, CD3/CD28 Stimulation and miRNA Transfection

For Treg polarization and CD3/CD28 stimulation studies, naïve lymph nodes were harvested and processed into single-cell suspensions. CD4^+^ T cells were purified using EasySep PE Positive Selection Kit (Stemcell Technologies, 18557). CD4^+^ T cell purity was routinely >90% as verified through flow cytometry. Cells (1 × 10^6^) were plated in 12-well plates in complete medium supplemented with plate-bound anti-mouse CD3ε, clone 145-2C11 (3 µg/mL) in the presence of anti-mouse CD28, clone 37.51 (3 µg/mL). For studies examining only CD23/CD28 stimulation, cells were harvested 48 h after plating for downstream analysis. For Treg polarization, cells were plated with recombinant mouse IL-2 (5 ng/mL) and recombinant human TGF-β1 (5 ng/mL) or recombinant TGF-β2 (5 ng/mL) (R&D Systems, Minneapolis, MN, USA) where indicated, in addition to the aforementioned amounts of CD3 and CD28. Five days after plating, cells were harvested for downstream analysis, and cell culture supernatants were collected for enzyme-linked immunosorbent assays (ELISAs). All cytokines were purchased from Biolegend (San Diego, CA, USA). In both experiments, cells were transfected with either 25 nM miR-466a-3p mimic (UAUACAUACACGCACACAUAAGA), 100 nM miR-466a-3p inhibitor (UAUACAUACACGCACACAUAAGA), or 25 nM scramble control, using HiPerfect Transfection Reagent from Qiagen (Valencia, CA, USA). Transfection efficiency was validated using quantitative real-time (qRT)-PCR.

### iTreg Generation

CD4^+^ T cells from BL6 FoxP3^GFP^ mice were purified using EasySep PE Positive Selection Kit (Stemcell Technologies, 18557). Cd11c^+^ allogeneic APCs were isolated from the spleens of C_3_H mice using EasySep PE Positive Selection Kit. The cells were cocultured for 3 days at a ratio of 5:1, T cells: APCs. In addition, anti-CD3ε (10 µg/mL), anti-CD28 (4 µg/mL), and IL-2 (5 ng/mL) were added to all wells and TGF-β1 (5 ng/mL) and TGF-β2 (5 ng/mL) were added where indicated. Cells were cocultured for 72 h before being collected for downstream analysis or sorted for purity and injected intravenously.

### Alloantigen CoCulture

Naïve lymph node cells were harvested and processed through a 100-µm cell strainer to make single-cell suspensions. Cells (1 × 10^6^) were plated in the presence of 50 µg/mL of alloantigen or no antigen (control) in complete RPMI in 12-well plates for 10 days. Fresh medium was added on day 5, and LNA-based miRNA inhibitor (anti-miR-466a-3p, Exiqon, Denmark) and control LNA were added every 3 days at 50 ng/mL. After 10 days, cells were collected for downstream analysis, and cell culture supernatants were stored at −20°C before being analyzed by cytokine-specific ELISA for IFNγ, TNFα, IL-17A, total TGF-β1 (Biolegend, San Diego, CA, USA), and TGF-β2 (R&D Systems, Minneapolis, MN, USA).

### Antigen Preparation (Splenocyte Lysates)

C_3_H and C57BL/6 mice were euthanized and their spleens were aseptically removed, homogenized, and passed through a 100-µm cell strainer to make single-cell suspensions in cold, serum-free media. Red blood cells (RBCs) were lysed, and the cell suspension was washed twice with cold serum-free media. Then, cells were re-suspended at a cellular density of 1 × 10^8^ cells/mL and subjected to four freeze (5-min liquid nitrogen)–thaw (10 min at 37°C water bath) cycles. Cells were then sonicated for 5 min, and the lysate was centrifuged at 350 *g* (10 min, 4°C) and the supernatant was recovered. The lysate was filtered with a 0.22-µm microporous membrane; protein concentration was determined using Qubit fluorometer (Thermo Fisher Scientific) and subsequently stored at 4°C.

### Graft-Infiltrating Cell (GIC) Extraction

Mice that received a skin transplant were sacrificed, and the transplanted graft was aseptically excised. Grafts were cut longitudinally, minced, and digested for 2 h at 37°C at 5% CO_2_ in PBS containing Type I collagenase (2.5 mg/mL) and hyaluronidase (0.25 mg/mL) (both from Sigma). Subsequently, GICs were obtained by spinning at 1,000 *g* for 7 min at 4°C before being re-suspended in FACS buffer and live cells enumerated using a hematocytometer, and either stained immediately for flow cytometry or plated overnight to recover GIC culture supernatants. Cell-free culture supernatants were recovered and stored at −20°C before being analyzed by cytokine-specific ELISA.

### Flow Cytometry and Antibodies

Relevant tissues were harvested and cells were homogenized and subsequently depleted of red blood cells as described above. To analyze immunophenotype surface markers, we stained single-cell suspensions using the recommended dilutions indicated on the manufacturer product sheets and gated them on PE-conjugated anti-CD4 (GK1.5) or FITC-conjugated anti-CD8α (53–6.7) where indicated. Antibodies used for flow cytometric analysis (BioLegend, San Diego, CA, USA) include Fc block, PE, PE/Cy7, and antigen-presenting cell (APC)-Cy7-conjugated anti-CD4 (GK1.5), PE and BV421-conjugated CD304 (Neuropilin-1) (3E12), PE-conjugated anti-IL-17A (TC11–18H10.1) Alexa Fluor 488 and BV421-conjugated FoxP3 (MF-14), FITC-conjugated Helios (22F6), APC-conjugated IFNγ (XMG1.2), APC and PerCP-Cy5.5-conjugated LAP (TGF-β1) (TW7-16B4), FITC-conjugated CD8α (53–6.7), BV650-conjugated CD223 (LAG-3) (C9B7W), and Alexa Fluor 700-conjugated CD49b. PE-conjugated IL-10 (JES5-16E3), PE-conjugated GATA3 (16E10A23), FITC-conjugated T-bet (4B10), APC-conjugated CD62L (MEL-14), BV650-conjugated CD278 (ICOS) (DX29) [PE-conjugated CD44 (IM7)] and PE/Cy7- and BV786-conjugated CD25 (3C7). Antibodies against nuclear proteins were probed using True-Nuclear Transcription Factor Buffer Set (BioLegend, San Diego, CA, USA), and intracellular cytokine staining was performed using Fixation/Permeabilization Solution Kit (BD, San Jose, CA, USA). The stained cells were then assessed by flow cytometer (FC500; Beckman Coulter, Brea, CA, USA) or BD FACSCelesta (BD, San Jose, CA, USA), and the resulting data analyzed by Cytomics CXP software (Beckman Coulter), DIVA software, or FlowJo. Sorting of cells was performed using a BD FACSAria II (BD, San Jose, CA, USA). The gates were set following the exclusion of debris. In addition, we used positive and negative controls for the fluorophores used. The events were displayed as a dot plot or as a contour map to show the relative intensity of scatter patterns. The gates were set at around populations of cells with common characteristics such as forward scatter, side scatter, and the density of marker expression.

### miRNA Expression Profiling

Draining lymph node CD4^+^ T cells purified to >90% purity using EasySep PE Positive Selection Kit (Stemcell Technologies, 18557) were subject to total RNA isolation using miRNeasy kit (Qiagen, Valencia, CA, USA), following manufacturer’s protocol. The concentration and purity of the isolated RNA were determined using a spectrophotometer, and the integrity of the RNA was verified using Agilent Eukaryote Total RNA Nano Series II on an Agilent 2100 BioAnalyzer (Agilent Tech, Palo Alto, CA, USA). Only samples with an RIN value above 8 were used for subsequent processing. The profiling of miRNA expression from samples was performed using the Affymetrix GeneChip miRNA 4.0 array platform (Affymetrix, Santa Clara, CA, USA) at the Johns Hopkins Deep Sequencing and Microarray Core (http://www.microarray.jhmi.edu/) following the manufacturer’s protocol. This array version covers all mature miRNA sequences in miRBase Release 20 (http://www.mirbase.org/). The stained chip was scanned on a GeneChip Scanner (Affymetrix) and microarray image data were analyzed using Affymetrix Power Tools (APT) to generate Robust MultiArray Average (RMA) values as well as detection above background (DABG) *P*-values as well as for normalization and quality control of data. Hybridization signals that showed aberrant properties and were <3 standard deviations over the mean background value were excluded. Statistical significance (*P*-values) for “detection calls” was determined by Affymetrix test. Probe sets with a *P*-value lower than 0.05 were called present (true). The log-transformed fluorescence intensity values were mean-centered and visualized by heat maps. miRNA fold changes were obtained from the array, and miRNAs with only a greater than 1.5-fold change were considered for further analysis. Predicted miRNA targets, alignments, and mirSVR scores were determined using online miRNA databases:microrna.org, and TargetScan Mouse 6.2. Ingenuity Pathway Analysis (IPA) (Qiagen, Valencia, CA, USA) was used to identify the molecular and functional annotations and canonical biological pathways potentially influenced by target genes of differentially expressed miRNA. The array data were deposited into the Genome Expression Omnibus (GEO) of NCBI (https://www.ncbi.nlm.nih.gov/geo/) and can be accessed *via* accession number GSE109160.

### Immunoblotting

Cell extracts were collected using RIPA lysis buffer supplemented with sodium orthovanadate, PMSF, and protease inhibitor (Sigma). Protein concentration was measured using Qubit fluorometer (Thermo Fisher Scientific) and was subjected to gel electrophoresis and transferred onto a nitrocellulose membrane. Blots were blocked with 5% BSA in TBST, washed, and probed overnight at 4°C with antibodies against TGFβ2 (1:1,000, R&D Systems, MAB73461), TGFβR3 (1:2,000, R&D Systems, AF5034), Smad2/3 (1:1,000, CST, 5678), Phospho-Smad2 (Ser465/467)/Phospho-Smad3 (Ser423/425), (1:1,000, CST, 8828), Smad4 (1:1,000, CST, 38454), and Phospho-Smad4 (Thr276), (1:1,000, Thermo Fisher Scientific, PA5-64712). The next day, blots were washed in TBST and then incubated at room temperature for 1 h with a horseradish peroxidase-labeled secondary antibody. Following secondary antibody incubations, blots were washed multiple times with TBST, exposed to a chemiluminescent reaction, Pierce™ ECL Western Blotting Substrate (Thermo Fisher Scientific, Rockford, IL, USA), and were exposed to film. Optical densities of films were quantified (sample minus background) using ImageJ.

### Measurement of Cytokines

Cell culture supernatants from indicated *in vitro* experiments, GIC culture supernatants obtained *ex vivo*, or serum samples were analyzed for the following cytokines: IFNγ, TNFα, IL-17A, total TGF-β1, latent TGF-β1, and free-active TGF-β1, ELISA kits were purchased from Biolegend (San Diego, CA, USA). For the detection of TGF-β2, ELISA kit was purchased from R&D Systems (Minneapolis, MN, USA).

### RNA Extraction and qPCR

CD4^+^ T cells in the dLN or spleens of grafted mice were purified using EasySep PE Positive Selection Kit (Stemcell Technologies, 18557), and total RNA was isolated using miRNeasy kit (Qiagen, Valencia, CA, USA), following manufacturer’s protocol. The expression of indicated mRNA and miRNA levels was determined by qRT-PCR. The quality and amount of RNA was investigated using Nanodrop 2000 (Thermo Fisher Scientific, Rockford, IL, USA). For miRNA expression analysis, cDNA was made from total RNA using miRNA cDNA Synthesis Kit, with Poly(A) Polymerase Tailing (ABM, Canada, G902). Two steps with hot-start miRNA qRT-PCR were carried out using EvaGreen miRNA Mastermix (ABM, Canada, MasterMix-mS) with mouse primers for SNORD96A (control), miR-466a-3p, miR-466e-3p, miR-466p-3p, miR-15a-5p, miR-181c-5p, miR-27a-3p, and miR-19b-3p (ABM, Canada). Expression levels were normalized to SNORD96A. For mRNA expression analysis, cDNA was made from total RNA using miScript cDNA synthesis kit from Bio-Rad (Hercules, CA, USA). A two-step amplification with a 60° annealing temperature for qRT-PCR was carried out using SsoAdvanced™ SYBR^®^ green supermix from Bio-Rad (Hercules, CA, USA) with mouse primers for TGFβ1, TGFβ2, TGFβR3, PTEN, FoxP3, Smad2, Smad3, TGFβR1, and TGFβ3. All qRT-PCR experiments were carried out on a CFX96 (or 384) Touch Real-Time PCR Detection System (Bio-Rad, Hercules, CA, USA). Expression levels were normalized to β-actin mRNA levels. Fold changes were calculated using the 2^−ΔΔCT^ method. Primers are detailed below.

**Table:** 

Gene	Primer	Sequence (5′–3′)	Accession Number
β actin	Forward Reverse	GGCTGTATTCCCCTCCG CCAGTTGGTAACAATGCCATGT	NC_000071.6
PTEN	Forward Reverse	TGGATTCGACTTAGACTTGACCT GCGGTGTCATAATGTCTCTCAG	NM_008960.2
FoxP3	Forward Reverse	CCCATCCCCAGGAGTCTTG ACCATGACTAGGGGCACTGTA	NM_054039.2
TGF-β3	Forward Reverse	AACAGCCACTCACGCACAGTG GCACAACGAACTGGCTGTCTG	NM_009368.3
TGF-β2	Forward Reverse	CTTCGACGTGACAGACGCT GCAGGGGCAGTGTAAACTTATT	NM_009367.4
TGF-βR1	Forward Reverse	TCTGCATTGCACTTATGCTGA AAAGGGCGATCTAGTGATGGA	NM_009370.3
TGF-βR3	Forward Reverse	GGTGTGAACTGTCACCGATCA GTTTAGGATGTGAACCTCCCTTG	NM_011578.4
TGF-β1	Forward Reverse	GAGAAGAACTGCTGTGTGCG GTGTCCAGGCTCCAAATATAGG	NM_011577.2
Smad2	Forward Reverse	ATTCCAGAAACGCCACCTCC GCTATTGAACACCAAAATGCAGG	NM_010754
Smad3	Forward Reverse	GCGTGCGGCTCTACTACATC GCACATTCGGGTCAACTGGTA	NM_013095.3

### Hematoxylin and Eosin (H&E) Staining

Grafts were excised and fixed by immersion in 4% paraformaldehyde in PBS, overnight. Fixed tissues were embedded in paraffin, sectioned, and stained with H&E. Color bright field images and picture montages were taken using a Cytation-5 Imaging Reader (BioTek Instruments, Winooski, VT, USA).

### Statistical Analysis

Prism 6 and 7 software (Graphpad) were used for statistical analysis. In skin-graft experiments, we used groups of at least seven mice. Data were depicted as means ± SEM. Student’s *t*-test was used to compare data between two groups. One-way ANOVA with a Tukey *post hoc* test was used to compare three or more groups. A log-rank (Mantel–Cox) test was used to determine the significance of survival curves. A *P* < 0.05 was considered to be significant.

## Results

### dLN Treg Cell Response to Allograft

**Figure 1:**
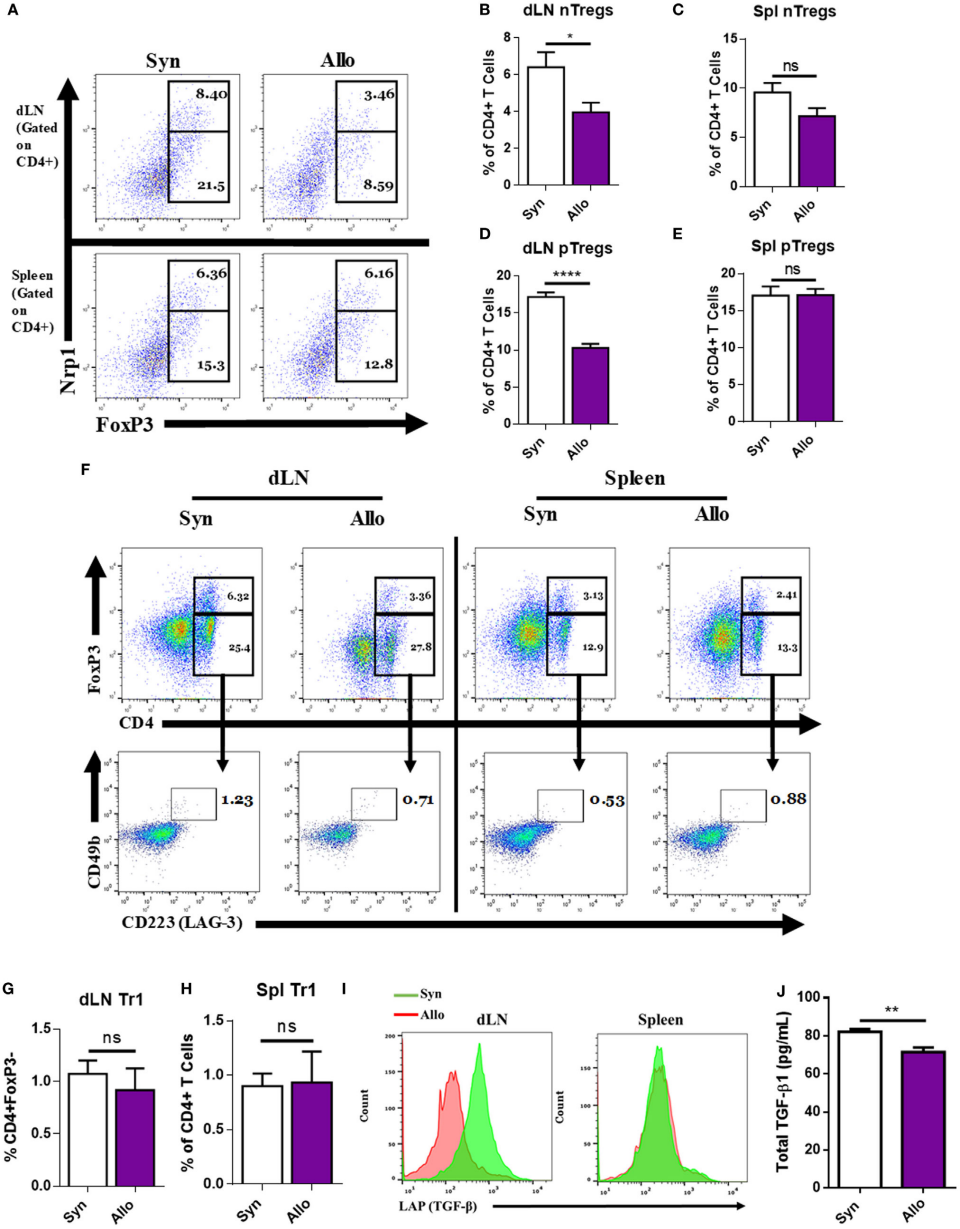
Allografting alters the draining lymph node (dLN) regulatory T cell (Tregs) phenotype. Ten days after syn- or allografting, mice were sacrificed and organs of interest were harvested. dLNs and spleens were analyzed for Treg cell phenotype by flow cytometry. **(A)** Representative flow cytometry dot plots gated on CD4^+^ cells, displaying the percentage of natural Tregs (nTreg) present through coexpression of CD4, FoxP3, and Nrp1 and the percentage of peripheral Tregs (pTreg) that are CD4 and FoxP3 positive, and Nrp1^LO^ or negative. **(F)** Dot plots (lower panel) gated on CD4^+^, FoxP3^−^ cells (upper panel), displaying CD223 (LAG-3), CD49b double-positive Tr1 cells. **(I)** Overlaid histograms gated on CD4^+^FoxP3^+^ cells, displaying LAP expression. **(B,C,D,E,G,H)** Quantification of flow cytometry results. **(J)** Enzyme-linked immunosorbent assay of total transforming growth factor-beta1 (TGF-β1) in the serum of mice on the day of sacrifice. *n* = 12 (syngeneic) or 18 (allogeneic) mice per group. Data are presented as mean ± SEM of three independent experiments. *P < 0.05, **P < 0.01, ****P < 0.0001 by Student’s *t-*test.

Regulatory T cells play a critical role in tolerance, and the decrease in their functions is associated with strong inflammation (3–8). To investigate the potential mechanisms that dampen the basal Treg induction during an immune response, we used an allogeneic skin-graft model of transplantation. To that end, C57BL/6 mice (H-2^b^, BL6), were given age- and sex-matched syngeneic (syn) (BL6) or allogeneic (allo) C_3_H (H-2^k^, C_3_H) full thickness ~1 × 1 cm^2^ tail skin transplants on the dorsal lateral surface. Ten days after transplantation, mice were sacrificed and their dLNs and spleens were harvested and assessed for the type and frequency of Tregs present. Among the main Treg subtypes, we investigated natural Tregs (nTregs), which are demarcated by surface CD4^+^ and Neuropilin-1 (Nrp1) expression, and expressed the transcription factor FoxP3 (27–29), pTregs that are CD4^+^, FoxP3^+^, Nrp1^−^, or Nrp1^LO^ (29, 30), and Tr1 T cells, which are CD4^+^, FoxP3^−^, CD25^−^, CD49b^+^, Lag-3^+^ (CD223^+^), and express inducible T cell costimulatory (ICOS). In addition, these cells express higher latent-associated TGF-β and secrete IL-10 (27, 31). In the dLN of allografted mice, but not in the spleen, there was a significant reduction in the percentage of nTregs and pTregs when compared to syngrafted mice (Figures 1A–E). By contrast, there were no significant changes in the percentages of Tr1 cells (Figures 1F–H). In addition, when looking at the amount of latent-associated peptide, TGF-β1 (LAP) on CD4^+^FoxP3^+^ cells, we observed a notable decrease in the percentages of these Tregs in the allograft dLN (Figure 1I). Due to the requirement of TGF-β1 for pTreg induction and the decrease in LAP on Tregs after allotransplantation, we looked at TGF-β1 levels in the serum on the day the mice were euthanized and found that its presence was diminished after allotransplantation as well (Figure 1J).

### An miRNA Cluster Is Altered in dLN CD4^+^ Cells

We next investigated if changes in Tregs were associated with alterations in miRNA expression because miRNAs are known to regulate T cell differentiation and plasticity. To that end, we isolated total RNA from purified CD4^+^ T cells in the dLNs of syn- or allografted mice or naïve mice, pooled the RNA from mice in the same group, and performed an miRNA expression microarray as a preliminary screening tool using an *n* of 1 per group. Differential fold change expression of 3,164 miRNAs between the naïve, syn and allo groups was performed. A table was constructed of all the miRNAs from the array that displayed a 1.5- or greater fold change in the allo group compared the syn group, while also displaying changes between the allo and naïve group, most of which also displayed a fold change greater than 1.5, with miR-7648-3p being the sole exception (Table 1). A compelling finding was that 10 of the 27 miRNAs that were found to be upregulated in the allo group (compared to both naïve and syn groups) all came from the same cluster of miRNA that was contained in the 10th intron of the Polycomb group gene Sex combs on the mid-leg with four MBT domains-2 (*Sfmbt2*) on mouse chromosome 2, henceforth referred to as Chromosome 2 miRNA cluster (C2MC) (Table 1). C2MC has also been referred to as the miR-297-669 cluster (32–34).

**Table 1:** miRNAs found to be altered after microarray analysis in the allogeneic group compared to naïve and syngeneic group and their associated fold change.

Transcript ID	Allogeneic vs. Naive	Allogeneic vs. Syngeneic
Mmu-miR-1291	−1.940334	−2.6569
Mmu-miR-5112	−3.72864	−2.11819
Mmu-miR-6368	−2.4931	−2.02958
Mmu-miR-7011-5p	−2.10665	−1.9088
Mmu-miR-1894-3p	−2.03285	−1.78943
Mmu-miR-6912-5p	−2.71246	−1.78591
Mmu-miR-6937-5p	−2.00668	−1.68382
Mmu-miR-6971-5p	−1.83436	−1.64353
Mmu-miR-7016-5p	−2.06684	−1.6174
Mmu-miR-7648-3p	−0.26363	−1.49674
Mmu-miR-324-3p	1.729733	1.5072
Mmu-miR-18a-5p	4.699893	1.531172
Mmu-miR-484	2.595994	1.551924
Mmu-miR-27b-3p	1.819848	1.631183
Mmu-miR-194-5p	1.819848	1.631183
Mmu-miR-181c-5p	6.726528	1.649867
Mmu-miR-128-3p	4.711249	1.682522
Mmu-miR-27a-3p	1.77744	1.685586
Mmu-miR-421-3p	3.317828	1.855922
Mmu-miR-19b-3p	2.20629	1.883595
Mmu-miR-192-5p	4.342631	1.888075
Mmu-miR-182-5p	2.459444	1.889462
Mmu-miR-let-7f-5p	5.910496	1.890165
Mmu-miR-30b-5p	1.916419	2.029614
Mmu-miR-30e-5p	2.124878	2.029614
Mmu-miR-21a-5p	1.916419	2.029614
Mmu-miR-30a-5p	2.191086	2.09029
Mmu-miR-15a-5p	3.724949	2.236029
Mmu-miR-466a-3p	2.332244	2.284712
Mmu-miR-466e-3p	4.058871	2.303314
Mmu-miR-466b-3p	3.908921	2.445917
Mmu-miR-466c-3p	2.474084	2.464956
Mmu-miR-466p-3p	2.452085	2.826219
Mmu-miR-669a-3p	2.601293	2.878352
Mmu-miR-669o-3p	2.129959	2.955524
Mmu-miR-467c-5p	2.129959	2.955524
Mmu-miR-467a-5p	2.97326	2.956093

### Validation of miR-466a Expression and Predicted mRNA Targeting

**Figure 2:**
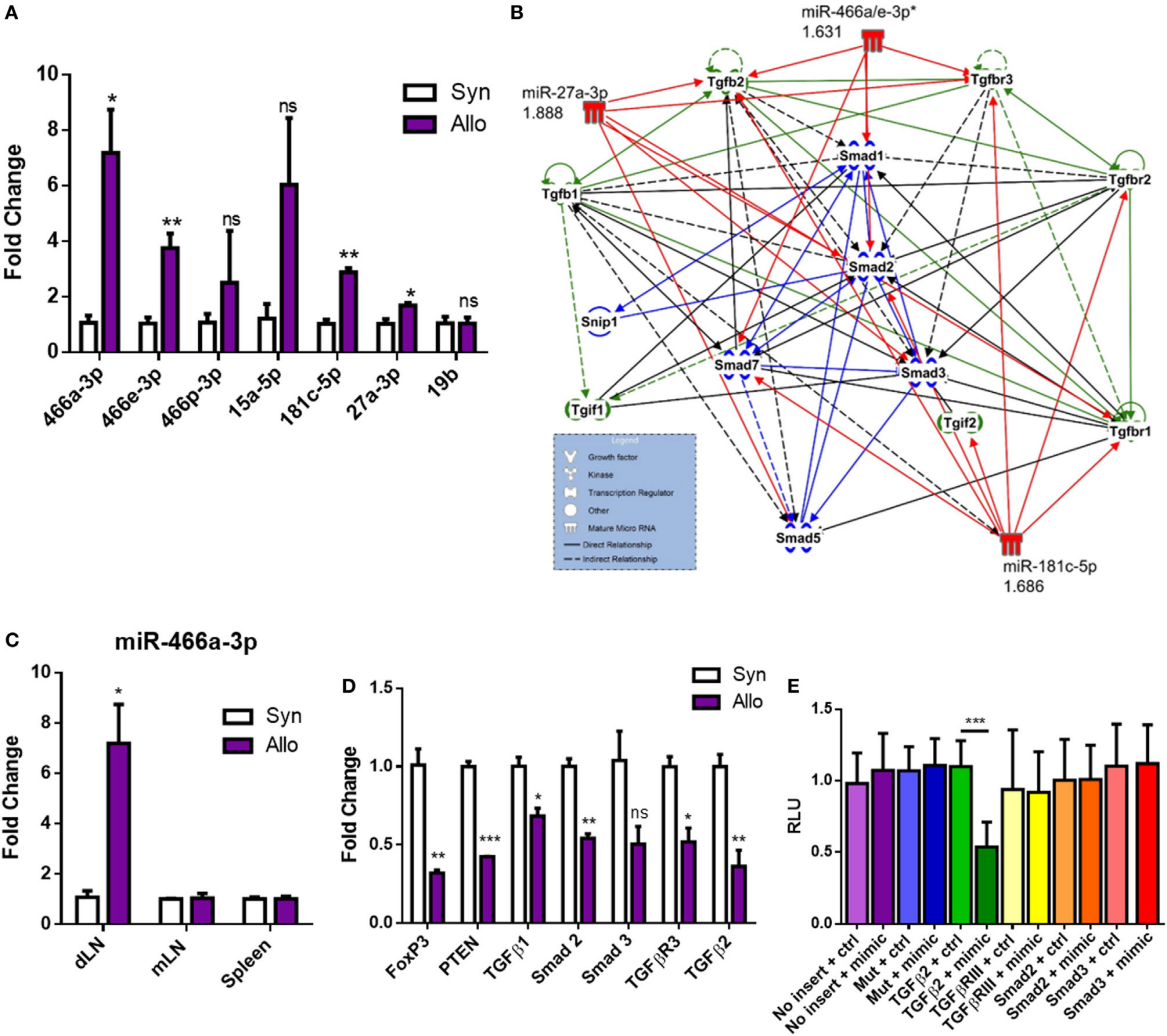
Alloantigen-induced microRNAs (miRNAs) target transforming growth factor-beta (TGF-β) family members and signaling molecules. Total RNA was extracted from purified CD4^+^ cells in the draining lymph node (dLN) of syn- or allografted mice 10 days post transplant. **(A)** Validation of microarray results through quantitative real-time (qRT)-PCR. **(B)** Ingenuity Pathway Analysis of seven of the top upregulated miRNAs and their predicted targets. **(C)** Fold change miR-466a expression in the dLN, spleen, or mesenteric lymph node (mLN) of purified CD4^+^ cells derived from syn- or allografted mice. **(D)** qRT-PCR validation of mRNA expression changes in the dLN CD4^+^ cells of syn- or allografted mice; *n* = 8 (syngeneic) or 12 (allogeneic) mice per group. **(E)** Relative luciferase expression in EL-4 cells transfected with luciferase reporter constructs which contained 3′ untranslated region (3′UTR) of proteins of interest or a mutated 3′UTR, together with miR-466a-3p mimics or the negative scramble control. A total of 48 h after transfection, luciferase activity was detected. Normalized data were calculated as the quotient of Renilla/firefly luciferase activities and are presented as mean ± SEM of three independent experiments with two technical replicates indicating six measurements. *P < 0.05, **P < 0.01, ***P < 0.005, ****P < 0.0001 by Student’s *t-*test.

Next, we validated the expression of these miRNAs through qRT-PCR (Figure 2A). Using TargetScan Mouse 6.2 and microrna.org, several of the miRNAs that were upregulated after allografting were found to target many members in the family of TGF-β signaling, consistent with a clear role for TGF-β1 in the differentiation of naïve CD4^+^ T cells into pTregs and tolerance (35–37). These miRNAs and their associated fold changes were input into IPA to display them alongside their predicted targets (Figure 2B). Our data indicating a decrease in pTregs, LAP^+^ Tregs, and circulating TGF-β1 (Figures 1A,D,I,J) suggested that the TGF-β1 pathway may be attenuated after allotransplantation, an avenue that was further pursued. Among the upregulated miRs, the specific miRNA from C2MC with the highest validated mean expression in CD4^+^ T cells draining from the allograft was miR-466a-3p (Figure 2A), henceforth referred to as miR-466a. This miRNA was chosen as the main miRNA of interest, both because of its noteworthy upregulation (Figure 2A) and because the seed sequence of miR-466a is identical to miR-297(a/b/c)-3p, miR-446d-3p, miR-467g, and miR-669d-3p, other members of C2MC. Upregulation of miR-466a after allotransplantation was specific to dLN CD4^+^ cells, as it was not significantly altered in splenic CD4^+^ cells or other peripheral LN CD4^+^ cells (Figure 2C). Predicted targets of C2MC in (Figure 2B) were validated through qRT-PCR (Figure 2D) to be downregulated in allograft dLN CD4^+^ cells. We next investigated the mRNA specifically targeted by miR-466a. Predicted target, binding, and miRSVR score of miR-466a–mRNA interactions are displayed in Table 2. We cloned the 3′ UTR of several mRNAs of interest (Smad2, Smad3, TGF-β2, and TGF-βR3) as well as a mutated 3′ UTR, immediately downstream of luciferase in a luciferase reporter assay. EL-4 cells were transfected with the luciferase reporters or a control vector lacking any 3′ UTR inserts in the presence of either a miR-466a mimic or a scramble control. We found that in the presence of miR-466a mimic, the luciferase activity of the reporter with the TGF-β2 3′ UTR cloned into its sequence was significantly lower, while such a decrease was not seen in the presence of the scramble control, any of the other cloned 3′ UTRs, or in the mutated control group (Figure 2E). This finding was consistent with the predicted 7mer-m8 seed match shared between miR-466a and TGF-β2.

**Table 2:** The predicted miRNA–mRNA binding interaction between indicated mRNA and miR-466a-3p, along with the associated mirSVR score for that interaction.

Predicted miRNA target	Predicted consequential pairing of miRNA (top) and mRNA target region (bottom)	mirSVR score
TGFβ2	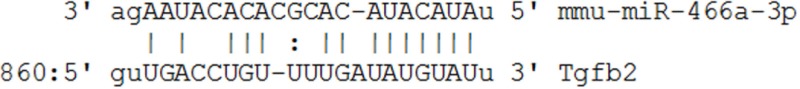	−0.5786
TGFβRIII	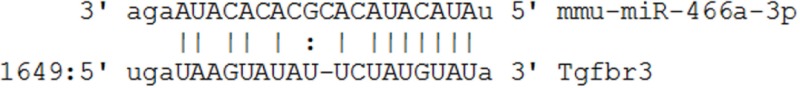	−0.0256
Smad2	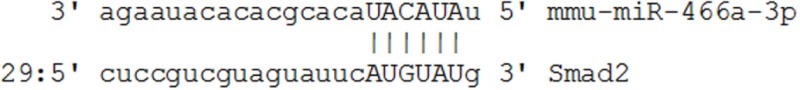	−0.0256
Smad3	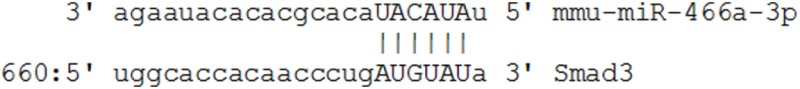	−0.3307

### miR-466a Targets Treg Polarization Through TGF-β2

**Figure 3:**
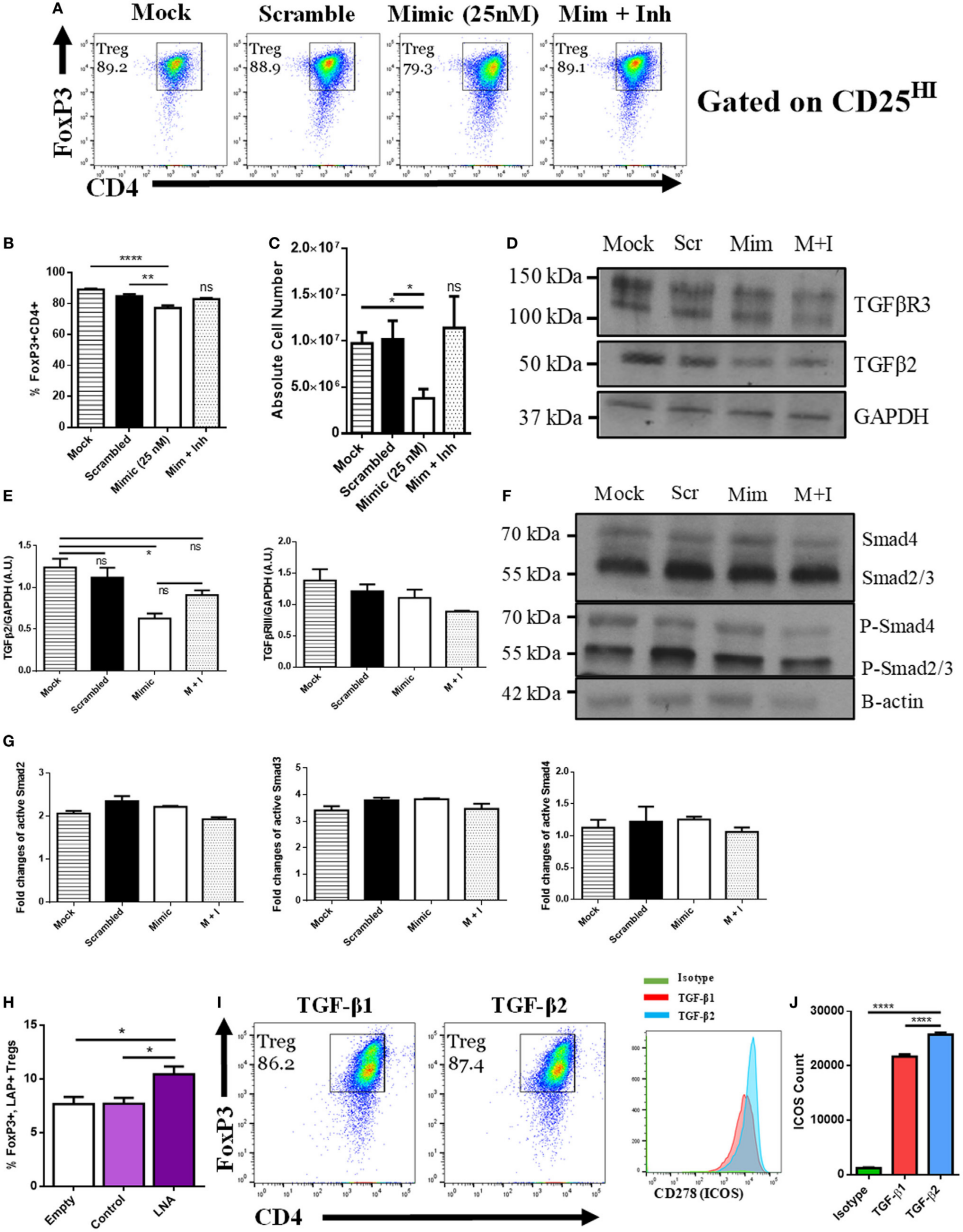
MicroRNA (miRNA) 466a-3p transfection inhibits regulatory T cell (Treg) polarization. Purified naïve CD4^+^ T cells were cultured under Treg-polarizing conditions along with the indicated mimic, control, or inhibitor conditions. Cells were harvested 48 h after addition of cytokines and miRNA mimics, inhibitors, or controls and subject to flow cytometry, immunoblot and quantitative real-time-PCR. The success of Treg polarization is examined as **(A)** representative dot plots gated on CD25HI cells and quantified in **(B,C)**. Representative immunoblots of indicated proteins are presented in **(D,F)**, along with associated densitometric measurements of transforming growth factor-beta 2 (TGF-β2) and TGF-βR3 **(E)**, and quantification of activated Smad 2, 3, and 4 **(G)**. CD4^+^ cells were purified from naïve mouse lymph nodes and stimulated *ex vivo* with CD3 (3 µg/mL) and CD28 (3 µg/mL) for 48 h and administered Locked Nucleic Acid or controls at the time of seeding. Quantification of flow cytometry data from LAP-expressing FoxP3 positive Treg cells. **(H)** Purified naïve CD4^+^ T cells were cultured with either TGF-β1 (5 ng/mL) or TGF-β2 (5 ng/mL), along with CD3 (3 µg/mL), CD28 (3 µg/mL), and IL-2 (5 ng/mL) for 5 days. **(I)** representative dot plots of FoxP3, CD4-positive Tregs gated on CD25HI, **(J)**, and their associated CD278 (ICOS) expression. Data are presented as mean ± SEM of three independent transfection experiments. *P < 0.05, **P < 0.005, ****P < 0.0001 by ANOVA with Tukey’s multiple comparison test.

To directly test the role of miR-466a on Treg differentiation, we used an *in vitro* Treg polarization model. To that end, purified naïve CD4^+^ T cells cultured with cytokines were transfected with either mock (empty vector), scramble control (25 nM), mimic (25 nM), or mimic + inhibitor (100 nM). The data showed that transfection with mimic, but not any of the other conditions, could suppress the generation of Tregs as demarcated by the coexpression of CD4 and FoxP3 (Figures 3A–C). It was worth noting that the mimic caused a robust decrease in the total number of Tregs generated in culture when compared to controls (Figure 3C). Transfection efficiency was validated with qRT-PCR (Figure S1A in Supplementary Material). We next quantified the mRNA and protein levels of the predicted targets of miR-466a after transfection and found that the mRNA expressions of Smad2, Smad3, TGF-β1, TGF-β2, and TGF-βR3 were all reduced after mimic transfection compared to the other conditions (Figure S1B in Supplementary Material). However, upon examining the protein level, although both Smad2 and Smad3 showed active phosphorylation, as is to be expected upon TGF-β signaling, the only protein examined whose levels were decreased after mimic transfection was TGF-β2 (Figures 3D–G). Continued Smad signaling despite the reduction in TGF-β2 is likely due to persistent signaling through TGF-β1. In addition, there was a decrease in the amount of free-active TGF-β1 in the cells transfected with miR-466a mimic, and this alteration was reversed with the addition of the inhibitor (Figure S1C in Supplementary Material).

Next, we examined the effect of miR-466a inhibition in a model wherein there was no exogenously administered TGF-β1. To that end, naïve CD4^+^ cells were purified and stimulated *in vitro* with anti-CD3/CD28 Ab in the presence of an LNA, designed specifically to inhibit miR-466a/b/c/d/e/p-3p (will be referred to as LNA-466), or a control that was designed not to target any known miRs (LNA-ctrl). Cells treated with LNA-466 exhibited an increase in the number of CD4^+^ CD25^HI^LAP^+^ FoxP3^+^ Tregs compared to controls (Figure 3H). To confirm that TGF-β2 can have a pronounced effect on Treg polarization, naïve CD4 cells were polarized with either TGF-β1 (5 ng/mL) or TGF-β2 (5 ng/mL). Both culture conditions induced the polarization of naïve T cells into Tregs, but TGF-β2-iTregs had an increased expression of inducible T cell costimulatory (ICOS), a marker of Treg fitness (38), compared to TGF-β1 iTregs (Figures 3I,J).

### miR-466a Inhibitor Decreases Pro-Inflammatory and Increases Anti-Inflammatory Cells After Coculture With Alloantigen

**Figure 4:**
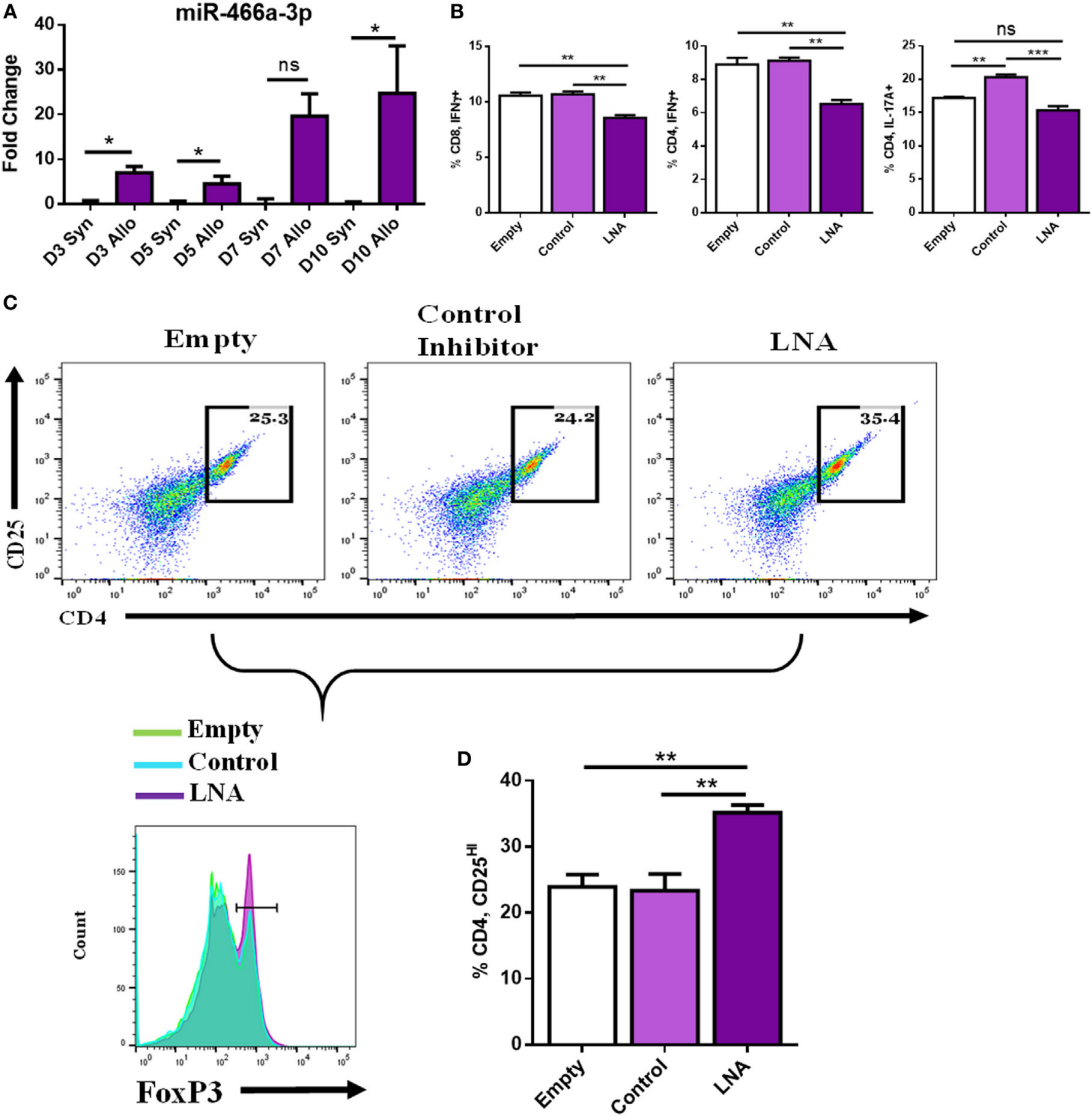
miR-466a inhibitor decreases pro-inflammatory and increases anti-inflammatory cells after coculture with alloantigen. Coculture of lymph node (LN) cells with alloantigen increases miR-466a-3p expression compared to LN cells cultured with syngeneic antigen at the indicated time points as determined by quantitative real-time-PCR **(A)**. LN cells were administered alloantigen (50 µg/mL) for 10 days in complete media. Fresh media and miRNA inhibitors or controls were added every 3 days. Cells were harvested and stained for Tc1, Th1, Th17, and Treg cells. **(B)** Quantitation of flow cytometry plots. **(C)** Histogram of FoxP3 expression (bottom row), gated on CD4^+^, CD25^+^ dot plots (top row), and quantified in **(D)**. Data are presented as mean ± SEM of two independent transfection experiments indicating six measurements. *P < 0.05, **P < 0.005, ***P < 0.001, ****P < 0.0001 by ANOVA with Tukey’s multiple comparisons test.

To mimic more closely the *in vivo* environment of transplantation, we implemented an *in vitro* coculture model wherein naïve LN cells were cultured with either syngeneic antigen or alloantigen (50 µg/mL). LN cells cocultured with syngeneic antigen died between days 3 and 5; however, LN cells cultured with alloantigen persisted and expanded. Coculture with alloantigen provoked a robust increase in the expression of miR-466a at several time points compared to cells cultured with syngeneic antigen (Figure 4A). Next, LN cells were cocultured with alloantigen and LNA-466 or control inhibitor and cells were analyzed by flow cytometry. LNA-466 addition resulted in a decrease in pro-inflammatory Th1 cells that were CD4^+^ IFNγ^+^, cytotoxic effector CD8^+^ IFNγ^+^ cells (Tc1), and CD4^+^ IL-17A^+^ Th17 cells (Figure 4B). In the same cultures, LNA-466 induced the increased proportions of CD4^+^CD25^HI^ cells and concomitantly increased FoxP3^+^ expression among that population, compared to controls (Figures 4C,D).

### LNA-466 Attenuates Inflammatory Markers After Allogeneic Skin Transplantation

**Figure 5:**
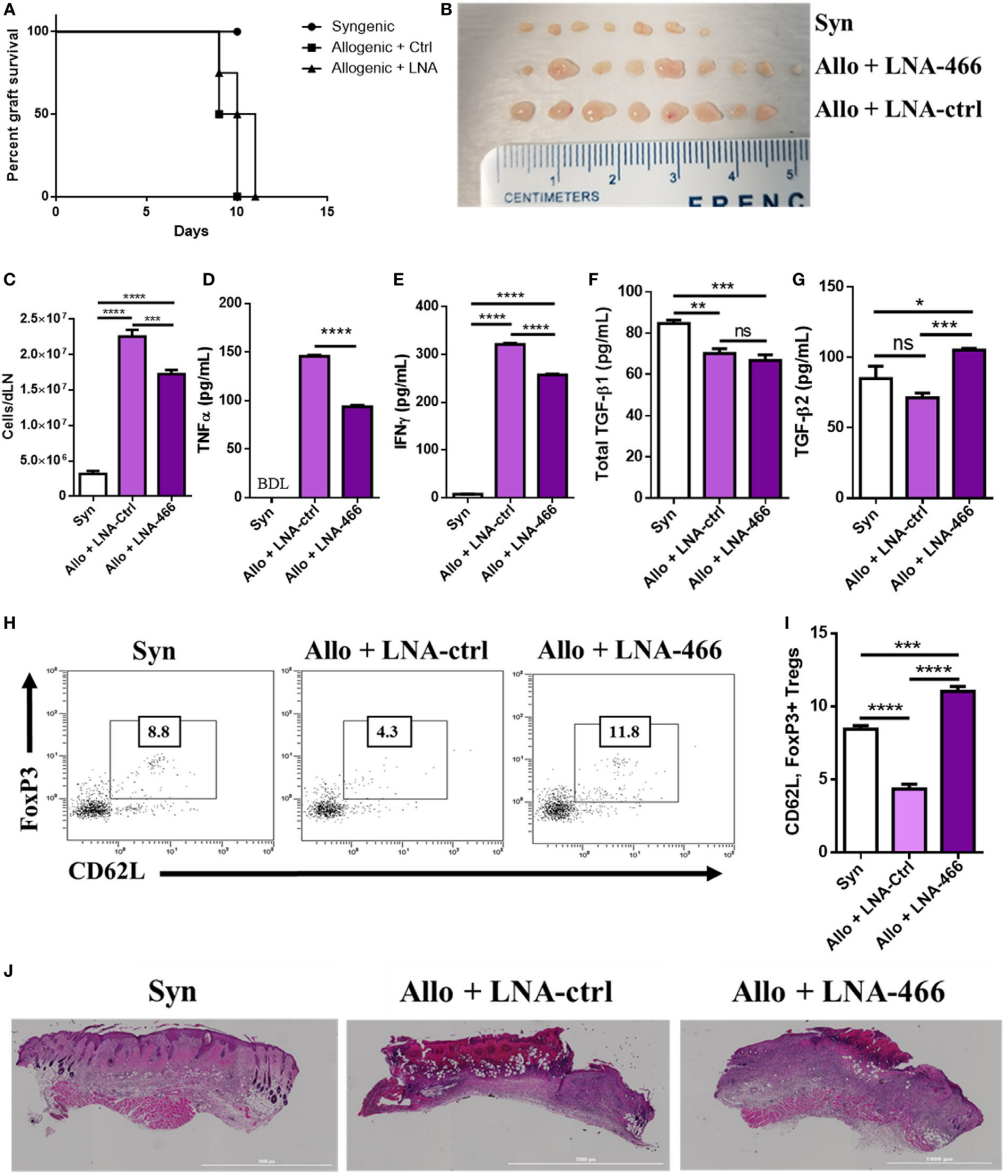
Locked nucleic acid (LNA) mitigates draining lymph node (dLN) effector cell and cytokines. Female C57BL/6 mice were given either syn (BL6) or allo (C_3_H) tail skin grafts. Mice receiving allografts were given either LNA-466 (10 mg/kg) or LNA-Ctrl (10 mg/kg) intraperitoneally 1 day before skin transplantation and every third day after that until termination of the study. **(A)** Survival curve of mice receiving skin transplants and indicated LNA or controls; *n* = 4 (syngeneic), 7 (allogeneic + Ctrl), or 8 (allogeneic + LNA). dLNs were harvested 12 days after skin transplantation from indicated groups imaged in **(B)**, and absolute cell counts were taken in **(C)**. dLN cells were plated overnight in complete media and culture supernatants were harvested and subjected to enzyme-linked immunosorbent assay (ELISA) for TNFα **(D)** and IFNγ **(E)**. Serum was taken upon sacrifice and subjected to ELISA for transforming growth factor (TGF)-β1 **(F)** and TGF-β2 **(G)**. Dot plots of circulating cells double positive for FoxP3 and CD62L, data are gated on CD4^+^ cells **(H)**, and quantified in **(I)**. **(J)** Hematoxylin & eosin stains of grafts excised from mice upon sacrifice. Data are presented as mean ± SEM; *n* = at least 4 per group. *P < 0.05, **P < 0.005, ***P < 0.001, ****P < 0.0001 by ANOVA with Tukey’s multiple comparisons test.

Because LNA-466 was effective in attenuating inflammatory T cells induced by alloantigen *in vitro*, we investigated its effect *in vivo*. To that end, C57BL/6 mice were given either C57BL/6 (syn) or C_3_H (allo) skin grafts and were administered LNA-466 or LNA-ctrl at a dose of 10 mg/kg starting 1 day before allografting and every third day thereafter, until termination of the study. While LNA-466 caused a slight delay in allograft rejection, it was statistically not significant (Figure 5A). However, mice given the allograft + LNA-466 did exhibit a significant decrease in the size and total cellularity of dLNs, thereby indicating a decreased host-versus-graft response and inflammation (Figures 5B,C). In the same experiment, LNA-466 failed to induce significant changes in the size and cellularity of the spleens (Figures S2A,B in Supplementary Material), thereby demonstrating that LNA-466 was targeting the dLNs, the primary site of immune response against alloantigen, and the site of mir-466a upregulation. To determine if LNA-466-mediated effect on Tregs was having a functional impact on inflammatory cytokines, dLN cells harvested from LNA-466-treated mice were cultured overnight and the supernatants were examined for cytokines. The data showed that LNA-466 derived cultures had significantly lower effector cytokines such as TNFα and IFNγ levels when compared to cells derived from allograft + LNA-Ctrl-treated mice (Figures 5D,E).

Mice receiving LNA-466 exhibited no significant changes in the amounts of circulating TGF-β1; however, consistent with the ability of miR-466a to target TGF-β2, the LNA-466 group demonstrated increases in circulating TGF-β2 when compared to syn or allograft + LNA-Ctrl groups (Figures 5F,G). Corroborating this finding, we found an increase in the number of circulating memory Treg cells (Figures 5H,I) in the LNA-466 group, surpassing the number of memory Tregs in the syn group.

When we performed histopathological analysis of the grafts, we noted that allograft + LNA-466 mice showed a decrease in the levels of cellular infiltration and graft damage compared to allograft + LNA controls (Figure 5J).

### LNA Reduces Intragraft Effector Cells and Cytokines

**Figure 6:**
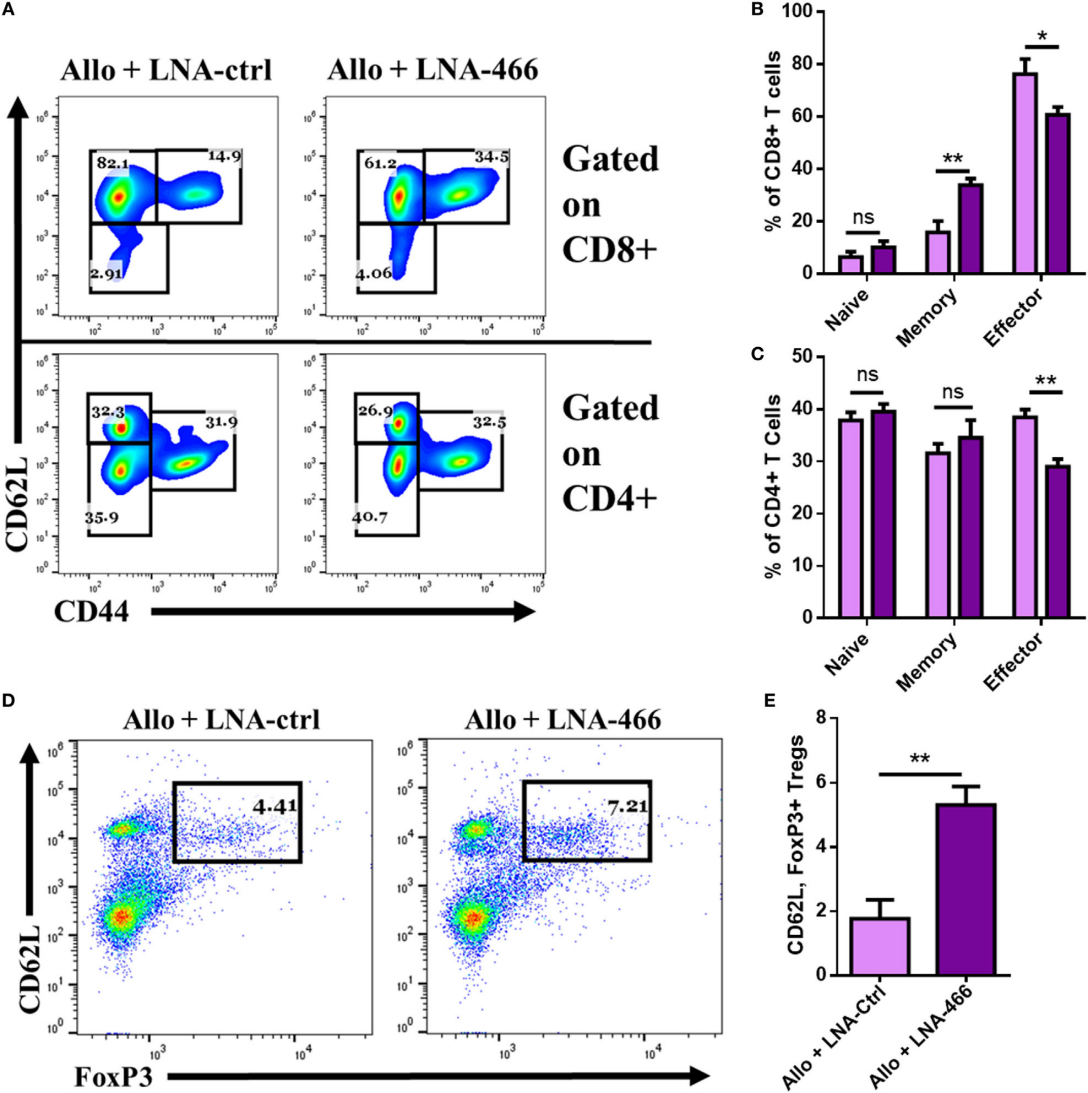

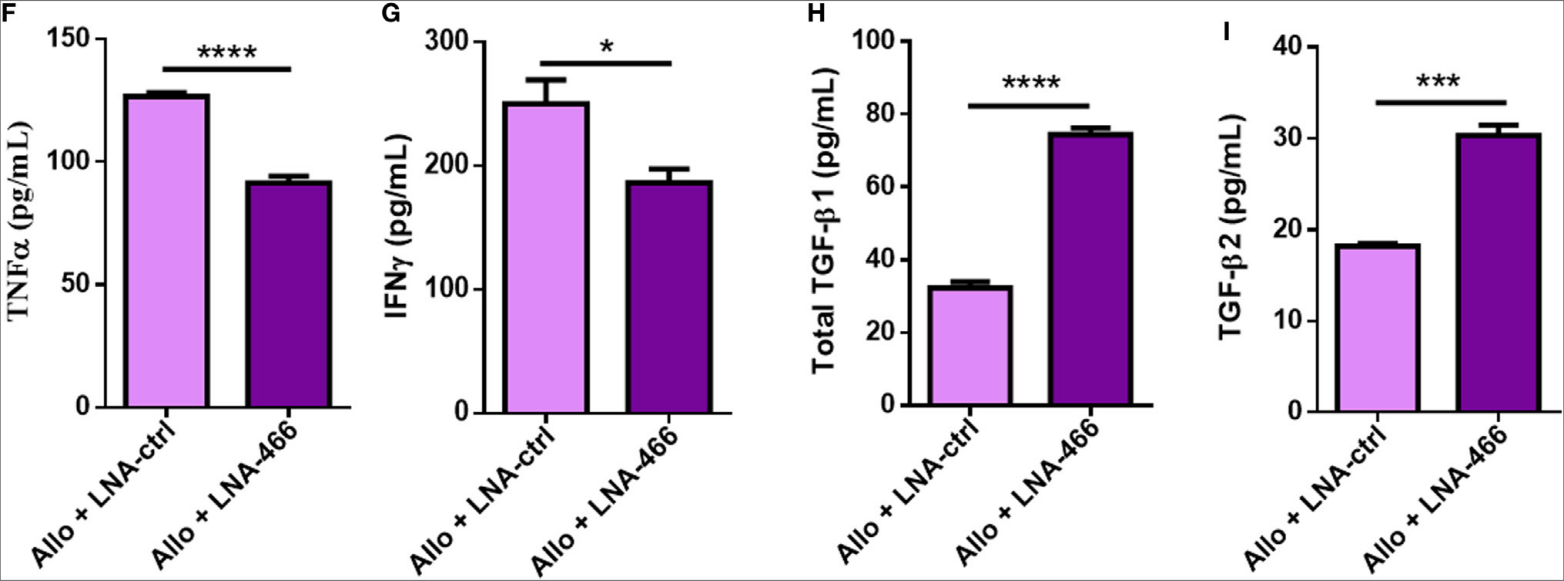
Locked nucleic acid (LNA) reduces intragraft effector cells and cytokines. Female C57BL/6 mice were given either syn (BL6) or allo (C_3_H) tail skin grafts. Mice receiving allografts were given either LNA-466 (10 mg/kg) or LNA-Ctrl (10 mg/kg) intraperitoneally 1 day before skin transplantation and every third day after that until termination of the study. Upon rejection, grafts were aseptically excised, minced, and enzymatically digested to dislodge graft-infiltrating cells (GICs). GICs were spun down, culture supernatants were collected, and live cells were used for flow cytometric analysis. Representative dot plots displaying naïve (CD62L^Low^, CD44^Neg^), memory (CD62L^+^, CD44^HI^), and effector (CD62L^Low^, CD44^HI^) cell types gated on CD8^+^ [**(A)**, upper row] and CD4^+^ [**(A)**, lower row] cells. Percentages for CD8^+^ and CD4^+^ are quantified in **(B,C)**, respectively. Dot plots of GICs double-positive for FoxP3 and CD62L, data are gated on CD4^+^ cells **(D)**, and quantified in **(E)**; *n* = 7 (allogeneic + Ctrl) or 8 (allogeneic + LNA). GIC supernatants were collected and subjected to enzyme-linked immunosorbent assay for the interrogation of effector cytokines TNFα **(F)** and IFNγ **(G)**, as well as anti-inflammatory cytokines transforming growth factor (TGF)-β1 **(H)** and TGF-β2 **(I)**. Data are presented as mean ± SEM. *P < 0.05, **P < 0.005, ***P < 0.001, ****P < 0.0001 by ANOVA with Tukey’s multiple comparisons test or a Student’s *t-*test.

Next, we directly studied the nature of cells and cytokines seen within the graft after LNA-466 or LNA-Ctrl treatment. To that end, the grafts were excised, minced, and digested to retrieve graft- GICs, which were either immediately stained, or plated for 24 h in complete media to obtain GIC culture supernatants. The data revealed that LNA-466-treated animals had a decrease in effector CD4^+^ and CD8^+^ GICs compared to the LNA-Ctrl group (Figures 6A–C). LNA-466 treatment also resulted in an increase in the percentage of graft infiltrating CD4, CD62L, FoxP3^+^ Tregs, compared to the LNA-Ctrl group (Figures 6D,E). Graft culture supernatants revealed that LNA-466 treatment led to reduced levels of effector inflammatory cytokines, TNFα (Figure 6F), and IFNγ (Figure 6G), as well as increases in total TGF-β1 and TGF-β2 (Figures 6H,I).

### TGF-β2-iTregs Are as Potent as TGF-β1-iTregs in Attenuating Allograft Rejection Response

**Figure 7:**
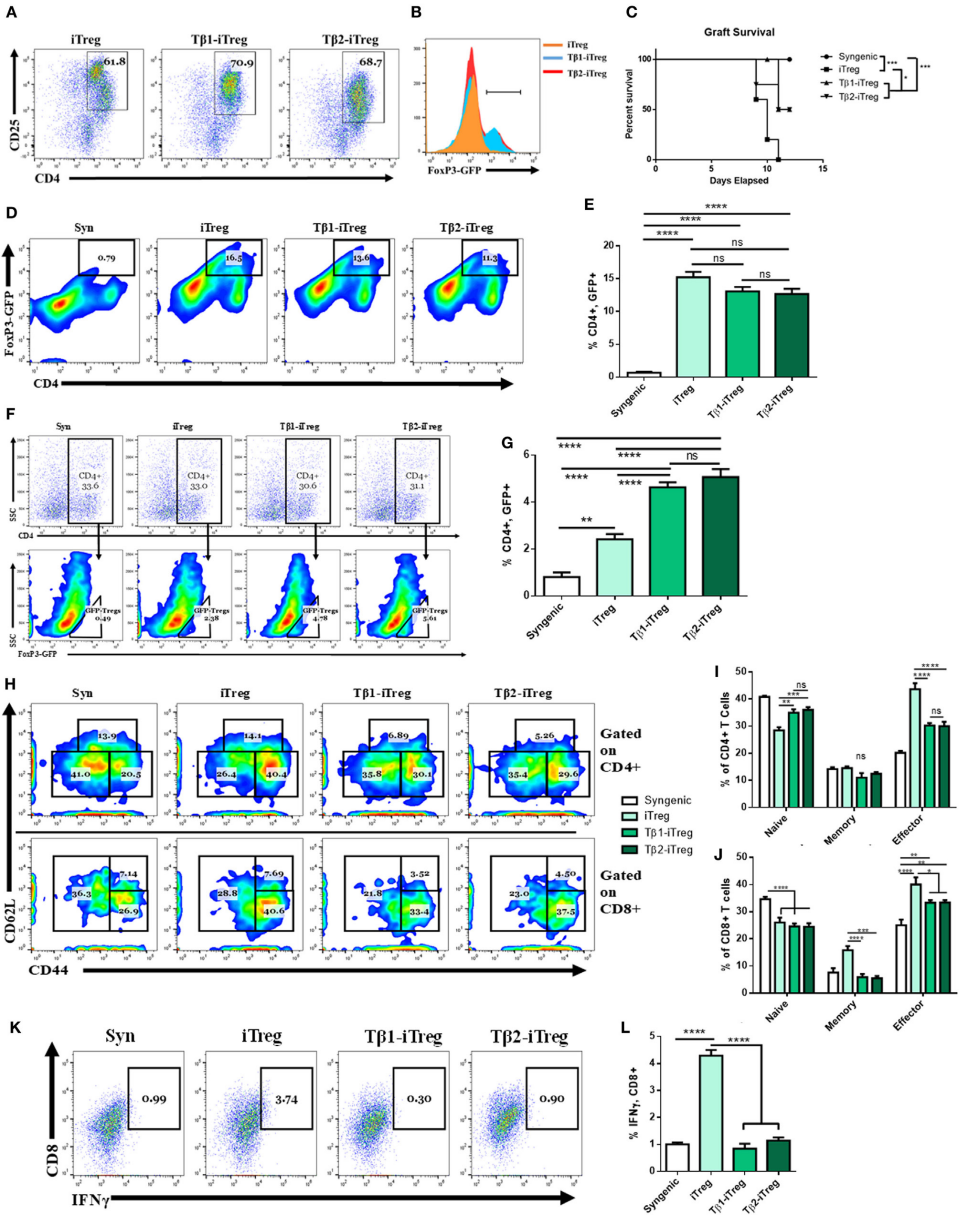
Transforming growth factor (TGF)-β2-induced regulatory T cells (iTregs) are equally as potent as TGF-β1-iTregs in ameliorating allograft rejection. CD4^+^ cells were purified from naïve BL6 FoxP3^GFP^ mice and cocultured with allogeneic splenic APCs along with anti-CD3ε (10 µg/mL), anti-CD28 (4 µg/mL), and IL-2 (10 ng/mL). Tβ1- and Tβ2-iTregs were also administered TGF-β1 (5 ng/mL) or TGF-β2 (5 ng/mL), respectively. Coculture proceeded for 3 days at which point the cells were either analyzed for FoxP3^GFP^ expression, or sorted into CD4^+^ FoxP3^GFP+^ cells and injected intravenously into graft-recipient mice 1 day before transplantation. **(B)** Histograms of FoxP3-GFP expression gated on dot plots of CD4^+^CD25^+^ cells in **(A)**. Female C57BL/6 mice were given either syn (BL6) or allo (C_3_H) tail skin grafts. Mice receiving allografts were administered 1 × 10^6^ iTregs intravenously 1 day before transplant. Grafts were scored starting 7 days after transplantation and continued until mice were sacrificed on day 12. Graft-infiltrating cells (GICs), draining lymph nodes (dLNs), and blood were collected for flow cytometric analysis. **(C)** Survival curve of indicated groups. **(D,F)** Pseudocolor plots of GFP^+^, CD4^+^ co-expressing iTregs in the **(D)** dLN and **(F)** among GICs. **(H)** Pseudocolor plots displaying graft-infiltrating CD4^+^ and CD8^+^ naïve, memory, and effector phenotypes. **(K)** Dot plots displaying IFNγ, CD8^+^ CTLs. Flow cytometry results quantified in **(B,E,G,I,J,L)**. *n* = 5 (iTreg), 10 (Tβ1-iTreg), or 9 (Tβ2-iTreg) mice per group. Data are presented as mean ± SEM. *P < 0.05, **P < 0.005, ***P < 0.001, ****P < 0.0001 by ANOVA with Tukey’s multiple comparisons test, or a log-rank (Mantel–Cox) test for the survival curve.

To study the role of TGF-β2-iTregs in suppressing inflammation, CD4^+^ cells from C57BL/6 FoxP3^GFP^ reporter mice were isolated and cultured with splenic APCs from allogeneic mice along with anti-CD3ε (10 µg/mL), anti-CD28 (4 µg/mL), and IL-2 (10 ng/mL). TGF-β1 is conventionally used to stimulate the production of both polyclonal and antigen-specific iTregs (4–8). Here, we tested the effect of culture with either TGF-β1 (5 ng/mL) or TGF-β2 (5 ng/mL) on iTreg generation. Similar to the polarization findings shown in Figure 3I, after 3 days of culture, we found that TGF-β2 was able to induce the generation of iTregs (Tβ2-iTregs) to the same extent and phenotype as TGF-β1 (Tβ1-iTregs) (Figures 7A,B). To test the efficacy of these cells at delaying acute rejection *in vivo*, after 3 days of coculturing, iTregs were sorted for CD4^+^, FoxP3^GFP^ coexpression, and 1 × 10^6^ cells were intravenously injected into allograft-recipient mice 1 day before skin transplantation. Syngeneic mice which did not receive any iTregs were used as controls. Tβ2-iTregs displayed potency equivalent to Tβ1-iTregs at delaying graft rejection (Figure 7C), preventing graft destruction at a rate greater than iTregs generated without the addition of TGF-β1 or TGF-β2. iTregs were verified to be present in the dLN (Figures 7D,E) to the same extent among all groups, although iTregs induced with TGF-β1 or TGF-β2 showed greater potential to home to the allograft (Figures 7F,G). Indeed, grafts harvested 12 days after allotransplantation that were derived from mice administered Tβ1-iTregs and Tβ2-iTregs showed a decrease in graft-infiltrating memory CD4^+^ cells (Figures 7H,I) and a decrease in the number of graft-infiltrating memory and effector CD8^+^ cells (Figures 7H,J). In the periphery, Tβ1-iTregs and Tβ2-iTregs reduced circulating CD4^+^ and CD8^+^ cells displaying a memory phenotype, but did not cause any change in circulating Tregs (Figures S3A–F in Supplementary Material). Lastly, Tβ1-iTregs and Tβ2-iTregs could significantly reduce the number of graft-infiltrating IFNγ-secreting CTLs compared to mice which received only iTregs (Figures 7K,L).

## Discussion

Regulatory T cell generation and administration is rapidly becoming a promising treatment option for patients undergoing end-stage organ failure (3–8). Because alloantigens, unlike conventional antigens, activate a large proportion of T cells and induce a strong inflammatory response, we considered using this model to study the impact of such activation on miRNA expression in T cells leading to the induction of pro-inflammatory T cells, while constraining Tregs. Our results provide a mechanistic perspective on how epigenetic shifts in CD4^+^ T cell miRNA expression can influence the generation of Tregs by the modification of TGF-β2 expression. Our data demonstrated an upregulation of many members of rodent miRNA cluster, C2MC, after allotransplantation in dLN CD4^+^ cells. Through pathway analyses, these miRNAs, and specifically miR-466a-3p, were predicted to target several members of the TGF-β-signaling family. We showed that miR-466a directly binds to the 3′ UTR of TGF-β2 through reporter luciferase assays. It should be noted that while miR-466a and its target were validated in this study, there were still several miRNAs picked up by the array that were not investigated further, and it is possible that such miRNAs may contribute to the complex inflammatory cascade perpetuating graft rejection.

Despite being an isoform of the widely studied TGF-β1 (13, 24, 25, 35–37, 39, 40), little information is known about the role of TGF-β2 in the immune system (39, 40). Indeed, most of the information concerning TGF-β2 is in regard to its role in the development and function of aorta (41), Loeys–Dietz syndrome (42), and cancer (43–45). Moreover, most studies performed involving TGF-β1 in immune cells have used TGF-βRII-deficient cells to highlight the role of TGF-β1; however, this also cancels out any potential TGF-β2 signaling. Thus, our findings that miR-466a regulates TGF-β2, which in turn plays a key role in the generation of Tregs, are novel. This was demonstrated conclusively in our study by altering TGF-β2 levels *via* transfection of CD4^+^ cells under Treg-polarizing conditions with miR-466a mimics, which led to a decreased generation of Tregs, while mimic inhibition reversed this effect. Furthermore, this reduction in Treg generation was associated with decreased mRNA levels of TGF-β family members and signaling molecules Smad2/3; however, at the protein level, while Smad2/3 showed consistent activation status between the groups, only TGF-β2 expression was altered upon mimic transfection. The persistence in Smad signaling despite changes in TGF-β2 expression is likely due to signaling through exogenously administered TGF-β1 in that model of *in vitro* polarization. Interestingly, we found TGF-β2 to be equally as effective as TGF-β1 at polarizing naïve CD4 cells *in vitro*, even conferring increased ICOS expression to the polarized Tregs, a marker of Treg fitness (38). To further corroborate the role of miR-466a in T cell differentiation, we used LNA in a coculture model to inhibit miR-466a expression and found that LNA-466 caused an increase in CD4^+^ CD25^HI^ FoxP3^+^ Tregs and a decrease in pro-inflammatory Th1 cells, CD8^+^ IFNγ^+^ cells (Tc1), and CD4^+^ IL-17A^+^ (Th17) cells. Mice bearing allograft and treated with LNA-466 exhibited a significant decrease in inflammation, an increase in FoxP3^+^ Tregs at the grafted site, and an increase in circulating TGF-β2 and circulating memory Treg cells. Together, the current study demonstrates for the first time that allografts induce miR-466a in CD4^+^ T cells which inhibits Treg differentiation through the suppression of TGF-β2. Our data suggest that *in vivo* modulation of miR-466a may constitute a novel approach to induce Tregs and thereby inhibit inflammation that is seen in a variety of clinical disorders.

Contrary to our hypothesis that *in vivo* inhibition of miR-466a using LNA-466 would result in delayed allograft rejection, our data showed that LNA administration failed to delay allograft rejection compared to controls. This may be because allografts activate a larger proportion of T cells compared to conventional antigens, thereby provoking a more robust inflammatory response, and the effect of LNA-466 may be too subtle to quell this pernicious immune response in a model with such a high degree of genetic mismatch. Moreover, in humans, HLA matching eliminates such strong host-versus-graft reactions, which are further controlled by immunosuppressive drugs. Nonetheless, LNA-466 administration significantly altered the immune cell environment toward a more anti-inflammatory phenotype, and this was without altering the circulating levels of TGF-β1. Despite TGF-β1 levels remaining stable in this experiment, we cannot discount the confounding role TGF-β1 may have in these studies. Future work utilizing TGF-β1^−/−^ knockout mice will need to be performed to accurately dissect the specific impact each TGF-β isoform has on the generation and maintenance of Tregs. Thus, the data presented in this study paint a picture of a complex inflammatory environment wherein the modulation of TGF-β2, specifically, *via* miR-466a downregulation can modify most greatly the inflammatory environment in circulation and within the allograft.

After demonstrating how changes in TGF-β2 levels could alter Treg generation, we investigated the role of TGF-β2 and compared it to its well-studied isoform, TGF-β1, in Treg induction and expansion. TGF-β2 was found to be as effective as TGF-β1 at inducing Tregs in an allogeneic coculture model. In addition, these Tβ2-iTregs demonstrated an ability to reduce allograft-directed inflammation at a rate comparable to Tβ1-iTregs and better than iTregs that were not cultured with TGF-β isoforms. This is a novel finding that surely warrants further work—to dissect the differences, if any, between Tregs generated *via* TGF-β1 or TGF-β2.

miR-466a-3p is a member of C2MC, one of the largest clusters of miRNAs, containing 71 miRNA genes (32–34). C2MC contains subclusters 297–466–467–699, many of which have been implicated in disease processes, from cell fate decision (46) and apoptosis (47) to aging in the heart (48); however, most members of the 466 subclusters have been implicated in immune regulation (46, 49). C2MC is under tight, temporal, and spatial regulatory control and exists in Sfmbt2, a region known to be imprinted (33). Sfmbt2 is expressed preferentially in the paternal allele in early embryos, and in later stage extra-embryonic tissue, while CpG islands spanning the transcriptional start site are differentially methylated on the maternal allele during embryogenesis (33, 34). The developmental regulation of C2MC is especially germane to this work, because the member of C2MC that was most highly upregulated after alloantigen exposure, miR-466a, was found to target TGF-β2, a protein that is also under considerable governance due to its extensive involvement in proper development (41, 42). Another possible mechanism that could be mediating the effects of TGF-β2 on the immune system, other than the direct effect we noticed on CD4^+^ cells, is the effect of TGF-β2 on APCs. In a model of experimental autoimmune uveoretinitis and anterior chamber-associated immune deviation, TGF-β2-treated APCs could provoke antigen-specific tolerance *via* Treg induction *in vitro* and *in vivo* (50, 51). This is an exciting progression and is concordant with our finding that increases in TGF-β2 levels occurred in tandem with increased levels of Tregs and more importantly that TGF-β2 can robustly induce Tregs in an alloantigen coculture model. We found that purified CD4^+^ T cells draining the allograft expressed heightened miR-466a; it is quite possible that these cells could be secreting their miRNAs in exosomes as a form of cell–cell communication in the dLN microenvironment to dendritic cells. Exosomal trafficking between CD4^+^ cells and DCs has been well documented, and if CD4^+^ cells were secreting miRNA-filled exosomes to decrease DC TGF-β2 expression, in accordance with the studies mentioned above, this could result in less Tregs being induced (52). Indeed, Wilson et al. found this form of paracrine exosomal signaling to be dominant in CD4^+^ cells, specifically in Treg cells (21). Interestingly, in that same study, it was found that several members of C2MC were among the top upregulated miRs in Treg-derived exosomes (21). Considering that clinical transplantation involves lesser HLA incompatibilities than used in our murine model, and due to the salutary effects that *in vivo* manipulation of miR-466a has on allograft rejection, we suggest that the miRNA management of TGF-β2 may constitute a therapeutic modality for allograft rejection or other inflammatory diseases—clearly additional studies are necessary to reinforce this point. Our studies also indicate a heretofore unrecognized role for TGF-β2 in the robust induction of Treg cells, an observation that could be an additional strategy for decreasing allograft rejection severity without resorting to global immunosuppression.

## Ethics Statement

This study was carried out in accordance with the recommendations of the University of South Carolina Institutional Animal Care and Use Committee. All mice were housed at the AAALAC-accredited animal facility at the University of South Carolina School of Medicine.

## Author Contributions

Conceptualization: WB, MN, and PN; methodology: WB; investigation: WB; writing-original draft: WB; writing-review and editing: WB, MN, and PN; funding acquisition: MN and PN; resources: MN and PN; supervision: MN and PN.

## Conflict of Interest Statement

The authors declare that the research was conducted in the absence of any commercial or financial relationships that could be construed as a potential conflict of interest.
