# Supplementary material for: miR-466a Targeting of TGF-β2 Contributes to FoxP3+ Regulatory T Cell Differentiation in a Murine Model of Allogeneic Transplantation
Source: Front Immunol. 2018 Apr 9;9:688. doi: 10.3389/fimmu.2018.00688 (PMC5900016; doi:10.3389/fimmu.2018.00688)
Supplement: Supplementary file 1 [file Presentation_1.PDF]

Supplementary Materials

miR-466a targeting of TGF-β2 contributes to FoxP3+ regulatory T cell differentiation in a murine model of allogeneic transplantation

William Becker[1], Mitzi Nagarkatti[1], and Prakash S. Nagarkatti*[1]

[1]Department of Pathology, Microbiology and Immunology, University of South Carolina School of Medicine, Columbia, SC 29208, USA

Supplemental Figure 1

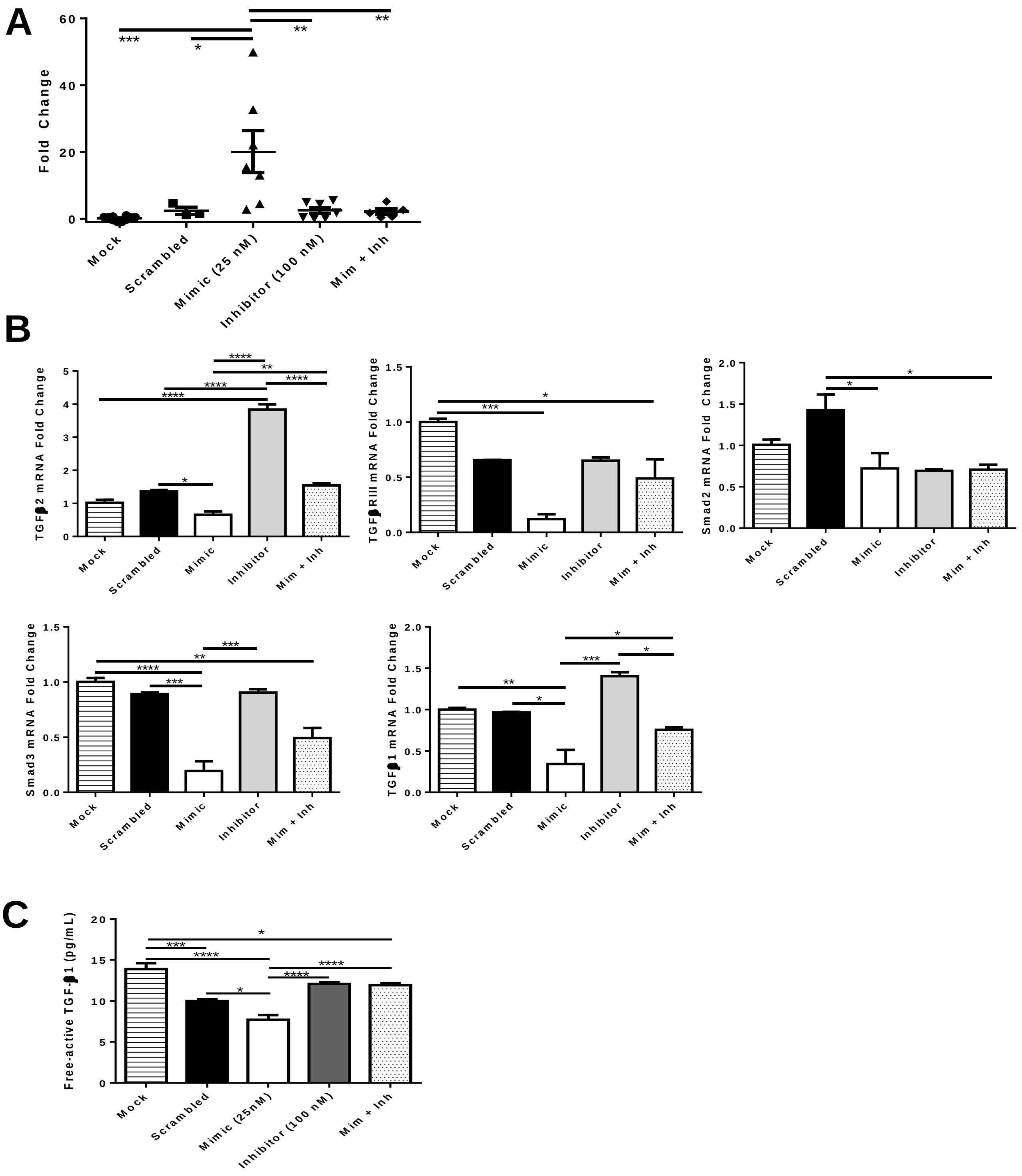

Supplementary Figure 1. *miRNA-466a-3p transfection in primary mouse CD4+ T Cells.* CD4+ T cells were isolated from naïve mouse LNs and purified using magnetic bead isolation. CD4+ cells were transfected with empty vector (mock), a scramble control, a miRNA-466a-3p mimic, or an inhibitor specific to miRNA-466a under Treg polarizing conditions for 48 hours in complete media before total RNA was harvested and cell supernatants were collected. Quantitative real-time PCR (qRT-PCR) of miRNA-466a-3p **A,** and indicated mRNAs **B.** ELISA of free-active TGF-β1 in the supernatants of indicated groups **C**. Data are presented as mean ± SEM of two independent transfection experiments indicating six measurements. *P<0.05, **P<0.005, ***P<0.001, ****P<0.0001 by ANOVA with Tukey's multiple comparisons test.

Supplemental Figure 2

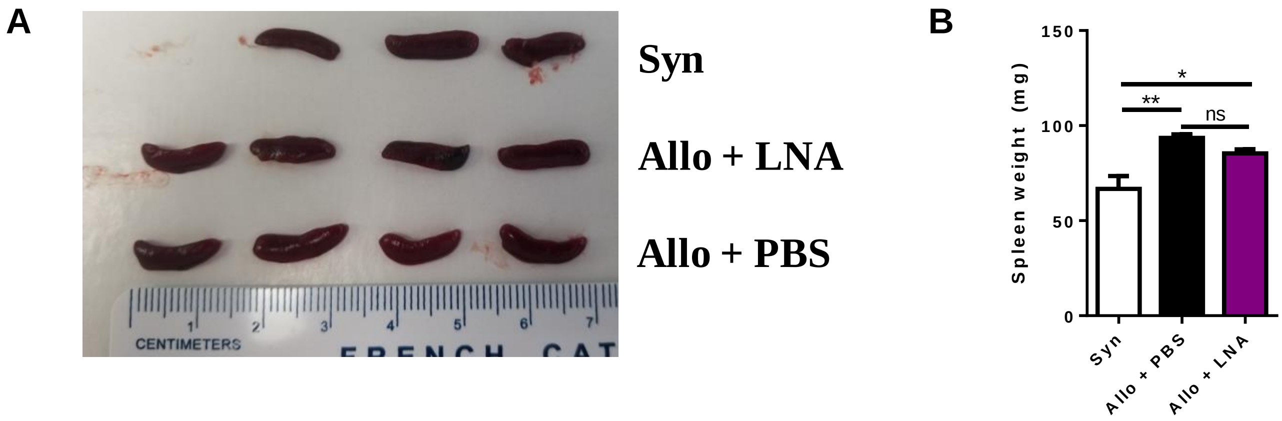

Supplementary Figure 2. *Splenic inflammation after skin transplantation*

Female C57BL/6 mice were given either syn (BL6) or allo (C_3H) tail skin grafts. Mice receiving allografts were given either LNA (10mg/kg) or PBS i.p. 1 day before skin transplantation, and every 3rd day after that until termination of the study. Image **A** and weight **B** of spleens harvested from mice in indicated groups upon rejection of allografts. Data are presented as mean ± SEM; n= at least 4 per group. *P<0.05, **P<0.005, ***P<0.001, ****P<0.0001 by ANOVA with Tukey's multiple comparisons test.

Supplemental Figure 3

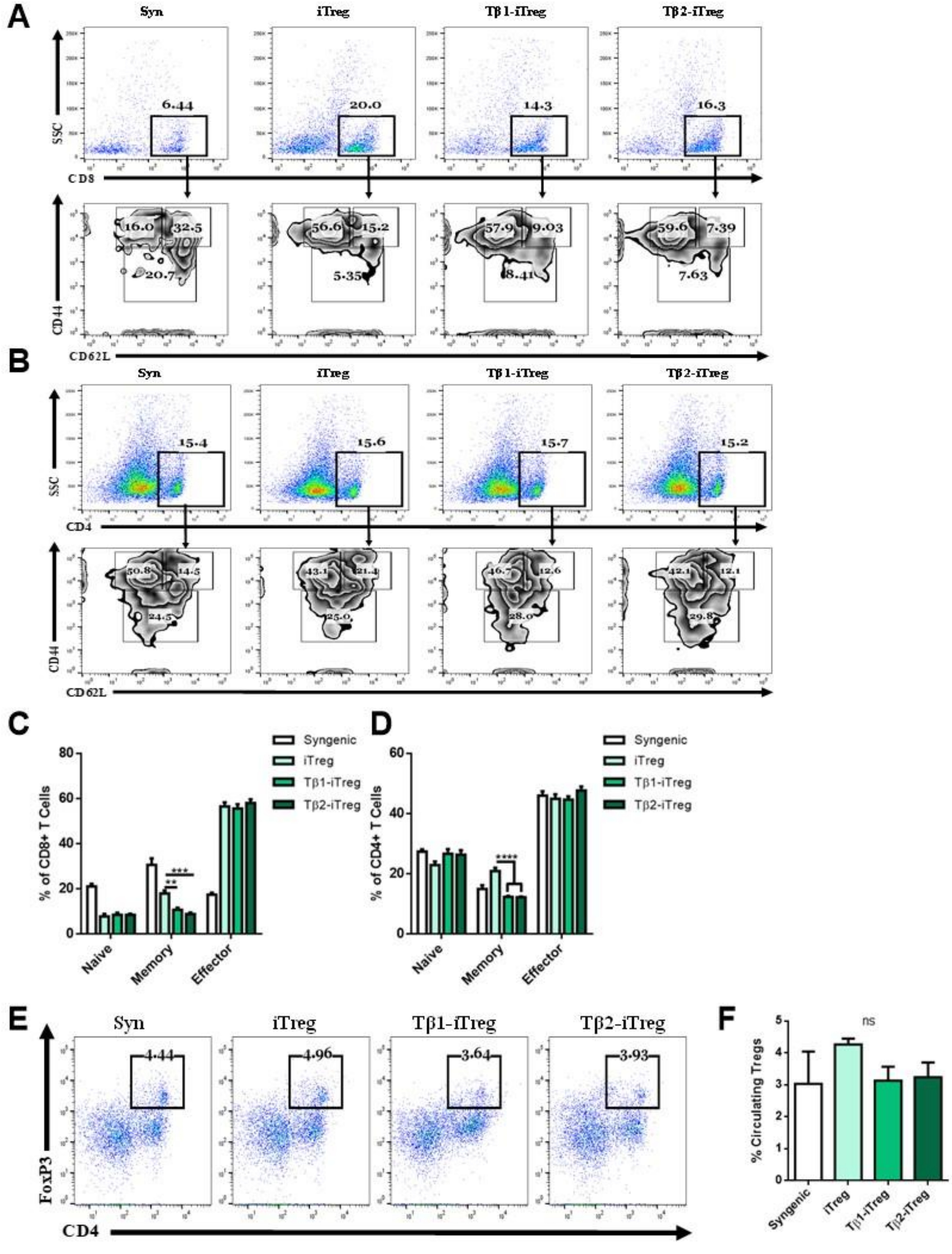

Supplementary Figure 3 *Circulating inflammatory profile after iTreg administration*

CD4+ cells were purified from naïve BL6 FoxP3^{GFP} mice and cocultured with allogenic splenic APCs along with anti-CD3ε (10μg/mL), anti-CD28 (4μg/mL) and IL-2 (10 ng/mL). Tβ1, and Tβ2-iTregs were also administered TGF-β1 (5ng/mL) or TGF-β2 (5ng/mL), respectively. Female C57BL/6 mice were given either syn (BL6) or allo (C_3H) tail skin grafts. Mice receiving allografts were administered $1x10^6$ iTregs intravenously 1d before transplant. 12 days post-transplant, mice were sacrificed and exsanguinated. **A, B** Zebra plots displaying naïve, memory and effector phenotypes, gated on CD4+ or CD8+ cells. **E** Dot plot of circulating Tregs. **C, D, F** Quantification of flow cytometry results. Data are presented as mean ± SEM; n= at least 4 per group. *P<0.05, **P<0.005, ***P<0.001, ****P<0.0001 by ANOVA with Tukey's multiple comparisons test.

Supplemental Figure 4

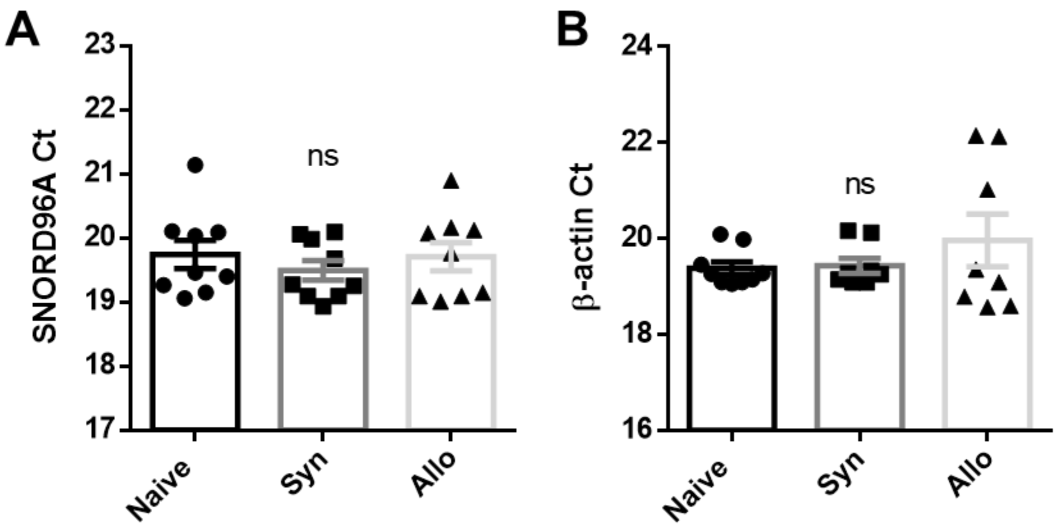

Supplementary Figure 4 *Validation of SNORD96A as miRNA and β-actin as mRNA reference genes*

CD4+ cells were purified from naïve BL6 mice or mice that received either syn (BL6) or allo (C_3H) tail skin grafts. RNA was extracted and quantitative real-time PCR (qRT-PCR) of **A** SNORD96A and **B** β actin was performed. Data are presented as mean ± SEM of three independent experiments indicating at least seven measurements and an ANOVA with Tukey's multiple comparisons test.
